# 16S rRNA PCR-Denaturing Gradient Gel Electrophoresis of Oral *Lactobacillus casei* Group and Their Phenotypic Appearances

**DOI:** 10.1155/2013/342082

**Published:** 2013-09-23

**Authors:** S. Piwat, R. Teanpaisan

**Affiliations:** ^1^Common Oral Diseases and Epidemiology Research Center, Faculty of Dentistry, Prince of Songkla University, Hat Yai 90112, Thailand; ^2^Department of Preventive Dentistry, Faculty of Dentistry, Prince of Songkla University, Hat Yai 90112, Thailand; ^3^Department of Stomatology, Faculty of Dentistry, Prince of Songkla University, Hat Yai 90112, Thailand

## Abstract

This study aimed to develop a 16S rRNA PCR-denaturing gradient gel electrophoresis (DGGE) to identify the species level of *Lactobacillus casei* group and to investigate their characteristics of acid production and inhibitory effect. PCR-DGGE has been developed based on the 16S rRNA gene, and a set of HDA-1-GC and HDA-2, designed at V2-V3 region, and another set of CARP-1-GC and CARP-2, designed at V1 region, have been used. The bacterial strains included *L. casei* ATCC 393, *L. paracasei* CCUG 32212, *L. rhamnosus* ATCC 7469, *L. zeae* CCUG 35515, and 46 clinical strains of *L. casei/paracasei/rhamnosus*. Inhibitory effect against *Streptococcus mutans* and acid production were examined. Results revealed that each type species strain and identified clinical isolate showed its own unique DGGE pattern using CARP1-GC and CARP2 primers. HDA1-GC and HDA2 primers could distinguish the strains of *L. paracasei* from *L. casei*. It was found that inhibitory effect of *L. paracasei* was stronger than *L. casei* and *L. rhamnosus*. The acid production of *L. paracasei* was lower than *L. casei* and *L. rhamnosus*. In conclusion, the technique has been proven to be able to differentiate between closely related species in *L. casei* group and thus provide reliable information of their phenotypic appearances.

## 1. Introduction

*Lactobacillus* strains are extensively used as probiotics in the food industry, and certain *Lactobacillus* species are also of importance in general health, providing a beneficial microflora in the oral cavity [1], intestinal tract [2, 3], and vagina [4]. The genus *Lactobacillus *contains a diverse assemble of Gram-positive, catalase negative, and nonsporulating, rod-shaped organisms and includes more than 140 species [5, 6]. Among those, *Lactobacillus casei *group, especially *L. casei, L. paracasei,* and *L. rhamnosus, *has attracted a lot of attention over the last 20 years. This is linked to the considerable economic importance of the *L. (para)casei* species, which is used in many food and feed applications such as dairy products and has a proven record in human and animal health. However, the taxonomy among the species in *Lactobacillus casei *group is still vague. The uncertain identification leads to confusion in phenotypic characters.

In recent years, the identification of lactobacilli evolving along with molecular methods based upon the 16S rRNA gene has been widely used. However, it is still difficult to differentiate unambiguously among these species. Many attempts to discriminate these species yielded inaccurate results, and only limited success could be achieved, largely due to the failure of differentiation between *L. paracasei* and *L. casei* strains [7 -10]. For example, most of the strains described as *L. casei* much more differ from the species type strain of ATCC 393^T^ than from *L. zeae* strains [11, 12]. In addition, *L. paracasei* strains and *L. casei* ATCC 334 are indistinguishable [13]. Thus, reliable and practical techniques to identify such strains are required. The technique of PCR-denaturing gradient gel electrophoresis (DGGE) has been introduced recently. This technique provides the information of variations in target genes within bacterial population which allows differentiating among the species that the DNA sequences differs in at least one base pair. In this study, it revealed that the DGGE method with the designed primers of the V1 and region of V2-V3 region of 16S rRNA genes enabled to distinguish among the species of *L. casei, L. paracasei, L. rhamnosus,* and *L. zeae*. In addition, the phenotypic characters of acid production and inhibitory effect on oral pathogen were analyzed.

## 2. Materials and Methods

### 2.1. Bacterial and Culture Conditions

Four reference strains of *Lactobacillus casei *ATCC 393^T^, *L. paracasei *CCUG 32212, *Lactobacillus rhamnosus* ATCC 7469^T^, and *L. zeae *CCUG 35515^T^ and 46 clinical strains, 10 *L. casei* isolates, 21 *L. paracasei* isolates and 15 *L. rhamnosus* isolates, were included in this study. All clinical strains were previously identified according to 16S-rRNA gene profiles by restriction fragment length polymorphism analysis (PCR-RFLP) and protein profiles by sodium dodecyl sulfate polyacrylamide gel electrophoresis (SDS-PAGE) [14]. The identification was also confirmed using sequencing of 16S rRNA genes.

### 2.2. Primers Used

 Two set of primers were used in this study, the first set of primers was HDA-1-GC (5′-**CGCCCGGGGCGCGCCCCGGGCGGGGCGGGGGCA** **CGGGGGG**ACTCCTACGGGAGGCAGCAGT-3′) and HDA-2 (5′-GTATTACCGCGGCTGCTGGCAC-3′) according to Walter et al. [10], which was designed targeting 200 bp of the V2-V3 region of 16S-rRNA gene. The second set of primers was CARP-1-GC (5′-**CGCCCGGGGCGCGCCCCGGGCGGGGCGGGGGCA** **CGGGGGG**GGCGTGCCTAATACATGCAA-3′) and CARP-2 (5′-GGCAGGTTACCCACGTGTT-3′), which was designed in this study targeting 112 bp of the V1 region. 

### 2.3. PCR-DGGE

All DNA samples were extracted using a Genomic DNA Extraction Kit (RBC Bioscience, Taipei, Taiwan), following the manufacturer's protocol for Gram-positive bacteria. The condition of PCR was that each 50 *μ*L PCR reaction contained 10 mM Tris-HCl pH 8.3, 50 mM KCl (GeneAmp PCR buffer II from Applied Biosystems, Foster City, CA, USA), 1 mM MgCl_2_ (Applied Biosystems), 0.1 mM each dNTP, 0.4 *μ*M both primers, 5 *μ*g/mL template, and 0.5 U Ampli-Taq DNA polymerase (Applied Biosystems). The PCR reactions were run using a GeneAmp PCR System 2400 (Applied Biosystems, Foster, CA, USA). For primers CARP-1-GC and CARP-2, reactions were run with an initial touchdown step in which the annealing temperature was lowered from 61 to 57°C in intervals of 2°C every 3 cycles, and 20 additional cycles were done with annealing at 55°C. Denaturation was performed at 95°C for 1 min, and extension was performed at 72°C for 1 min and 30 s. For primers HDA-1-GC and HDA-2, reactions were run for 35 cycles of denaturation at 95°C for 60 s, annealing at 56°C for 45 s, and extension at 72°C for 60 s. For both amplification cycles, an initial denaturation at 94°C for 5 min and a final extension at 72°C for 7 min were carried out.

### 2.4. DGGE Analysis

The Dcode Universal Mutation Detection System (BioRad, Hercules, CA, USA) was used for the sequence-specific separation of the PCR products. Electrophoresis was performed in a 8% polyacrylamide gel with gradient of 35 to 60% urea-formamide denaturant, and electrophoresis running time was adjusted to 6 h at 120 V. After electrophoresis, they were stained for 10 min in a SYBR Green solution (Molecular Probes, OR, USA) and analyzed under UV illumination. 

### 2.5. Inhibitory Effect of *Lactobacillus *against *Streptococcus mutans* ATCC 25175

The inhibitory effect of the *Lactobacillus* strains against *Streptococcus mutans* ATCC 25175 was assessed by an agar overlay method [15]. In brief, *Lactobacillus* strains (producer strains) were inoculated on the surface of the brain heart infusion agar and incubated anaerobically (80% N_2_, 10% H_2_, and 10% CO_2_) for 24–48 h at 37°C to develop visible macrocolonies. *S. mutans* ATCC 25175 was used as an indicator strain. The indicator strain was precultivated in the brain heart infusion broth (BHI), and the suspension of cells was adjusted to an optical density (OD) 0.25 at 600 nm. Thereafter, 5 mL of BHI soft agar (7 g/L agar) was seeded with 100 uL of an overnight culture of the indicator strain and immediately poured over the macrocolonies of *Lactobacillus*. The plates were incubated anaerobically at 37°C for 24 h to generate an inhibitory zone. The experiments were performed in triplicate.

### 2.6. Measurement of Acid Production

The acid production of the *Lactobacillus* strains was assessed according to Piwat et al. [16]*. Lactobacillus* strains were initially grown anaerobically (80% N_2_, 10% H_2_, and 10% CO_2_) to exponential phase in filter sterilized (pore size 0.22 *μ*m, Nalgene, NY, USA) de Man, Rogosa and Sharpe (MRS) broth (Lab M, Bury, UK) at 37°C for 16–18 h. Cells were harvested by centrifugation at 3000 rpm for 5 min at 4°C, washed twice in phosphate buffered saline (PBS; Oxoid, Basingstoke, UK), and inoculated into 50 mL fresh, prewarmed MRS broth containing 2% (w/v) glucose, pH 7.0, to give an optical density of 1.0 at 650 nm (corresponding to 10^10^ cells/mL). The cultures were then incubated in an anaerobic chamber (miniMacs Anaerobic Workstation, Don Whitley Scientific Ltd, UK) at 37°C.

Two milliliters of each sample was collected and analyzed for the growth and acid production at the start (0) and after 1.5, 3, 5, 7, and 24 h. Bacterial growth was determined by measuring the final OD reached at 650 nm, and the change in OD was calculated. In addition, the number of viable cells was also counted as CFU/mL on MRS agar following anaerobic incubation for 48 h. 

Acid production was studied by pH measurements using a pH electrode and pH-meter (Hanna pH 211, Hanna Instrument, UK). The amount of hydrogen ion [H^+^] was obtained from pH values according to the formula: [H^+^] = (10^pH^)^−1^.

The rate of acidification by each strain (acid production rate) was determined from the change in H^+^ (*δ*H^+^) divided by the average number of bacterial cells per hour in the logarithmic growth phase, as shown in the following equation:

(1) $$\begin{array}{c} \text{acid production rate}=\frac{\left(\delta {\text{H}}^{+}\right)}{\left[\left(\left(N_{2}-N_{1}\right)/2\right)*t_{2}-t_{1}\right]}, \end{array}$$

where *N*_1_ and *N*_2_ are number of bacterial cells at time point 1 (*t*_1_) and time point 2 (*t*_2_), respectively. The data presented are means of triplicate measurements, and all experiments were performed twice. 

## 3. Results 

**Figure 1:**
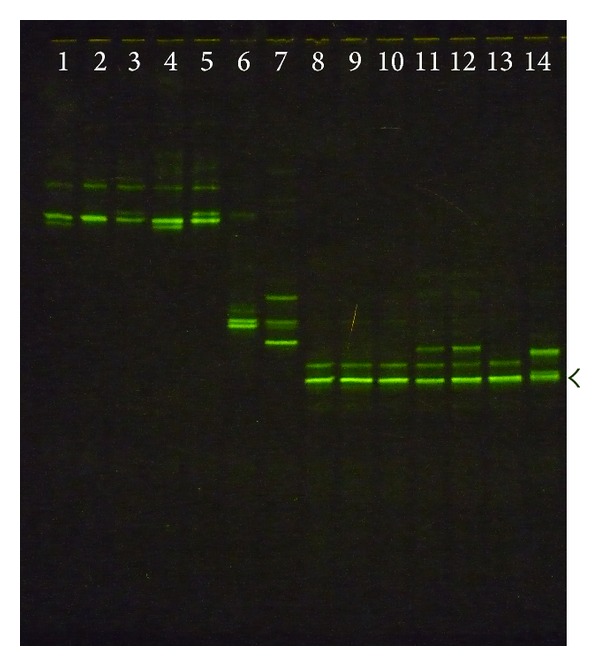
The DGGE patterns, V1 region of 16S rRNA, of *Lactobacillus casei *group in the 35–60% denaturant gel. Lane 1,* L. rhamnosus *ATCC 7469; Lanes 2–5, *L. rhamnosus *clinical isolates; Lane 6, *L. zeae *CCUG 35515; Lane 7,* L. casei *ATCC 393; Lanes 8–10, *L. casei *clinical isolates; Lane 11, *L. paracasei *CCUG 32212; Lanes 12–14, *L. paracasei *clinical isolates. The arrow indicates the major fragment of *L. casei *and *L. paracasei* strains.

**Figure 2:**
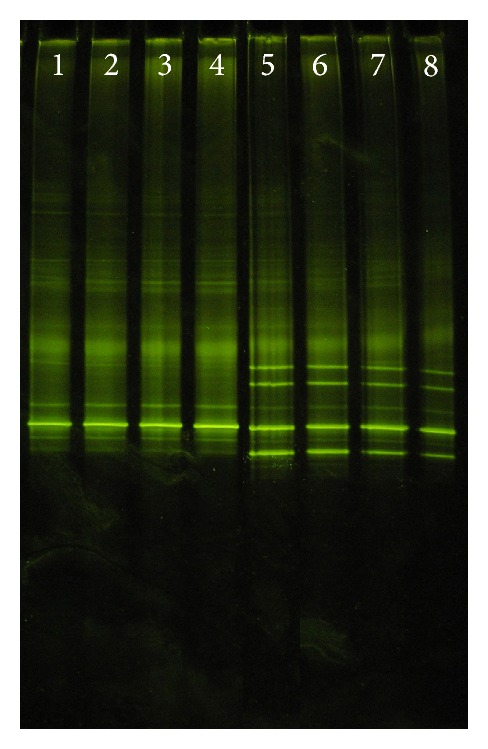
The DGGE patterns, V2-V3 region of 16S rRNA, of *Lactobacillus casei *group in the 35–60% denaturant gel. Lane 1,* L. casei* ATCC 393; Lanes 2–4, *L. casei *clinical isolates; Lane 5, *L. paracasei *CCUG 32212; Lanes 6–8, *L. paracasei *clinical isolates.

 The DGGE patterns of V1 region of 16S rRNA sequences are shown in Figure 1, and each type species strain and identified clinical isolate showed its own unique DGGE pattern. *L. rhamnosus *ATCC 7469 and identified clinical isolates revealed a different band from* L. zeae *CCUG 35515,* L. casei *ATCC 393, and *L. paracasei *CCUG 32212. It was noted that those of clinical strains identified as *L. casei* gave the different DGGE patterns from the type strain *L. casei *ATCC 393. All clinical strains identified as *L. casei*  had a major band with the same distance as* L. paracasei *strains; however, *L. paracasei *CCUG 32212 and most of clinical isolates identified *L. paracasei *showed the extraminor bands. When HDA1 and HDA2 primers were used to produce the DGGE patterns of V2-V3 region of 16S rRNA sequences, *L. casei *ATCC 393 showed the same DGGE pattern as all clinical strains identified as *L. casei. L. paracasei *CCUG 32212 and all clinical isolates revealed the same multiple bands which clearly differed from all *L. casei *strains (Figure 2).

**Figure 3:**
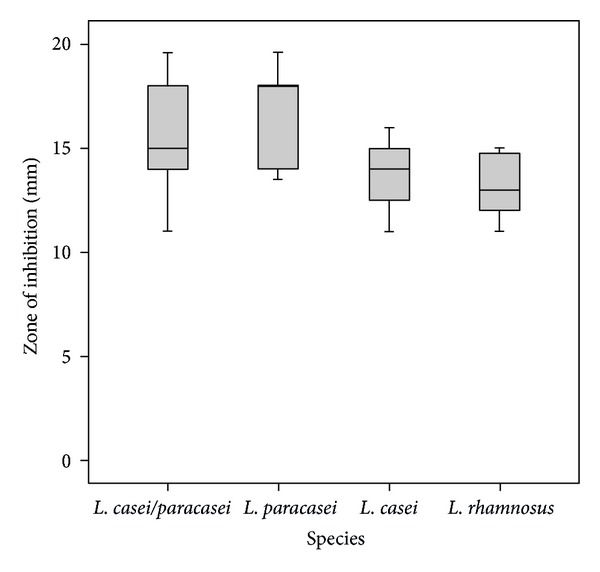
Inhibitory effects of clinical strains of *L. casei/paracasei/rhamnosus *against *S. mutans* ATCC 25175. Boxplot shows median, percentile (first and third percentile), and the minimum-maximum distribution of value.

 The ability for growth inhibition of *L. casei* group against *S. mutans* ATCC 25175 is shown in Figure 3. A statistically significant difference among the species was found (Kruskal-Wallis Test, *P* < 0.05) after *L. paracasei *strains were identified separately.* L. paracasei *strains had a significantly higher inhibitory effect than either *L. casei *or *L. rhamnosus* (Mann-Whitney *U* Test, *P* < 0.01).

**Figure 4:**
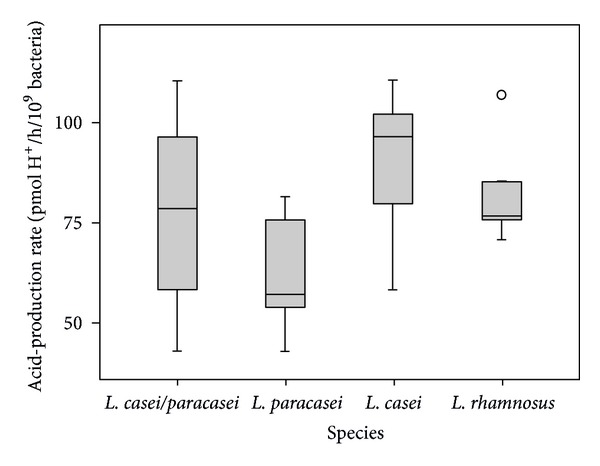
Acid-production rate of clinical strains of *L. casei/paracasei/rhamnosus *in the exponential growth phase. Results were calculated from the 1.5 to 5 h incubation period. Boxplots show median, percentile (first and third percentile), and the maximum-minimum values. Outlier values are presented as dots.

 Acid-production rate of clinical strains of *L. casei* group in the exponential growth phase was calculated from the 1.5 to 5 h incubation period. Also, a statistically significant difference among the species was found (Kruskal-Wallis Test, *P* < 0.05) after *L. paracasei *strains were identified separately. *L. paracasei *strains had a significantly lower acid-production rate than either *L. casei *or *L. rhamnosus* (Mann-Whitney *U* Test, *P* < 0.05) (Figure 4).

## 4. Discussion

The *Lactobacillus casei* group is one of special relevance for dairy food (cheese, yoghurt, and other fermented milk products) and pharmaceutical industry due to its important role in promoting human health. Based on their ability to inhibit the growth of various pathogens, they have been used as probiotics in the gut for decades [17]. Previously, a single species with five subspecies has been reclassified into three species including *L. casei, L. paracasei,* and *L. rhamnosus* in 1989 [18]. However, this classification initiated a controversy, generally due to the failure of differentiation between *L. paracasei* and former *L. casei *strains even by molecular technique [19 -21] including our previous study [14]. 

Research has been focused on the application of molecular biology techniques for accurate differentiation among the strains. We developed a DGGE method with the designed primers of the V1 in combination with V2-V3 region of 16S rRNA genes, which enabled to distinguish the close related strains in the *L. casei* group. It was shown that CARP1-GC and CARP2 primers could clearly distinguish the type strain *L. rhamnosus *ATCC 7469, *L. zeae *CCUG 35515,* L. casei *ATCC 393, and *L. paracasei *CCUG 32212 from each other, and each individual revealed its own unique pattern (Figure 1). It was surprising that those of clinical strains identified as *L. casei* gave different DGGE patterns from the type strain *L. casei *ATCC 393. The results indicated that there were different sequences in V1 region of all the type strains, which confirmed the information obtained by comparing the published 16S rRNA gene sequences of the type strain *L. rhamnosus *ATCC 7469, *L. zeae *CCUG 35515,* L. casei *ATCC 393, and *L. paracasei *CCUG 32212 [22]. 

HDA1-GC and HDA2 primers could distinguish the type strain and identified clinical isolates of *L. casei *from the type strain and identified clinical isolates of *L. paracasei *(Figure 2). By showing the multiple bands of all strains of *L. paracasei,* it indicated that *L. paracasei* strains contained more than one copy of 16S rRNA gene. This has been confirmed by DNA sequencing (data not shown). It is likely that the taxonomy of this group will undergo further changes with more extensive studies providing more evidence in the coming years. Thus, such method may assist in future taxonomic considerations of this group and could be a beneficial implementation for future research. 

In oral cavity, lactobacilli have frequently been isolated from carious lesions, and thus, they are thought to be associated with the development of dental caries. The reason was based on their acid production and aciduric characteristics [16, 23, 24]. As many *Lactobacillus* species have similar nutritional and growth requirements, it is often difficult to use classical microbiological methods to identify close related species. Consequently, in most of the dental literature, they are specified and are referred to only as lactobacilli. However, it is important to understand the certain role of various lactobacilli, whether they are harmful, beneficial, or neutral for the development of dental caries.

Nowadays, *Lactobacillus* was increasingly used as probiotic bacteria in the oral cavity [1]. The inhibitory activities of oral *Lactobacillus* against oral pathogens, for example, cariogenic bacteria, periodontopathogens, and *Candida, *have been reported [15, 25, 26]. However, the cariogenic characteristics of *Lactobacillus*, especially acidogenic activity, have always been concerned [27]. In most clinical studies, *L. casei* and *L. paracasei* have always been grouped together due to difficulties in their differentiation. They have been reported for their association with dental caries in several studies [28 -30]. However, less is known of how acidogenicity and inhibitory activity differ among these two species identified with current taxonomic methods. In this study, the heterogeneity of acid production and antimicrobial effect among the species of *L. casei/L. paracasei/L. rhamnosus *were clear when their identification was performed properly. It was found that *L. paracasei *strains were stronger in growth inhibition against oral pathogens and were weaker in acid production. The results indicated that *L. paracasei *may be of benefit as probiotics for the prevention of oral diseases than the others due to its low acidogenic and high inhibition effect.

In conclusion, the technique has been proven to be able to differentiate between closely related species in *L. casei* group and thus provide reliable information of their phenotypic appearances.
